# Pancreatic size and fat content in diabetes: A systematic review and meta-analysis of imaging studies

**DOI:** 10.1371/journal.pone.0180911

**Published:** 2017-07-24

**Authors:** Tiago Severo Garcia, Tatiana Helena Rech, Cristiane Bauermann Leitão

**Affiliations:** 1 Post-graduate Program in Medical Sciences: Endocrinology, Universidade Federal do Rio Grande do Sul (UFRGS), Porto Alegre, RS, Brazil; 2 Intensive Care Unit, Hospital de Clínicas de Porto Alegre, Porto Alegre, RS, Brazil; 3 Endocrine Division, Hospital de Clínicas de Porto Alegre, Porto Alegre, RS, Brazil; Baylor College of Medicine, UNITED STATES

## Abstract

**Objectives:**

Imaging studies are expected to produce reliable information regarding the size and fat content of the pancreas. However, the available studies have produced inconclusive results. The aim of this study was to perform a systematic review and meta-analysis of imaging studies assessing pancreas size and fat content in patients with type 1 diabetes (T1DM) and type 2 diabetes (T2DM).

**Methods:**

Medline and Embase databases were performed. Studies evaluating pancreatic size (diameter, area or volume) and/or fat content by ultrasound, computed tomography, or magnetic resonance imaging in patients with T1DM and/or T2DM as compared to healthy controls were selected. Seventeen studies including 3,403 subjects (284 T1DM patients, 1,139 T2DM patients, and 1,980 control subjects) were selected for meta-analyses. Pancreas diameter, area, volume, density, and fat percentage were evaluated.

**Results:**

Pancreatic volume was reduced in T1DM and T2DM vs. controls (T1DM vs. controls: -38.72 cm3, 95%CI: -52.25 to -25.19, I2 = 70.2%, p for heterogeneity = 0.018; and T2DM vs. controls: -12.18 cm3, 95%CI: -19.1 to -5.25, I2 = 79.3%, p for heterogeneity = 0.001). Fat content was higher in T2DM vs. controls (+2.73%, 95%CI 0.55 to 4.91, I^2^ = 82.0%, p for heterogeneity<0.001).

**Conclusions:**

Individuals with T1DM and T2DM have reduced pancreas size in comparison with control subjects. Patients with T2DM have increased pancreatic fat content.

## Introduction

The pancreas plays a key role in diabetes mellitus, a progressive disease characterized by chronic hyperglycemia [1] in the context of insulin resistance [2] and/or beta cell dysfunction and death [3]. Beta cell loss secondary to apoptosis leads to a reduction in beta cell mass [4, 5]. Although islets of Langerhans represent only 1% of the total pancreas, autopsy studies have demonstrated reduced pancreas size in both type 1 [6] and type 2 diabetic subjects [7].

Insulin deficiency and the lack of a trophic effect of insulin on acinar cells[6]as well as the chronic inflammation associated with insulitis [8, 9]may explain the reduction in pancreas size in type 1 diabetes (T1DM), whereas atherosclerosismight play a role in type 2 diabetes (T2DM) [10, 11]. In both type 1 and type 2 diabetes, pancreatic size reduction may also be associated to exocrine pancreatopathy[12, 13]. However, the reduction in pancreatic size may also be the cause, and not a consequence of diabetes [14, 15].

Imaging studies are expected to produce reliable information regarding pancreas size. However, while some studies using ultrasound (US), computed tomography (CT), and magnetic resonance imaging (MRI) to assess pancreas size in diabetes have shown reduced pancreatic size in individuals with diabetes as compared to controls [16–18], no differences were observed in others [19, 20]. Such inconclusiveness may be related to the small sample size of most studies evaluating this issue.

Interestingly, CT and MRI are widely used to measure liver steatosis [21, 22], which is closely related to obesity and diabetes [22]. More recently, imaging protocols have produced accurate non-invasive measurements of pancreatic fat content in humans [23, 24]. Excess ectopic fat storage has been linked to insulin resistance [22], and pancreatic fat content has been negatively associated with insulin secretion [25].

The aim of the present study was to systematically review the literature and synthesize data regarding pancreatic size and fat content in diabetes using meta-analysis.

## Methods

### Data sources and searches

**Fig 1:**
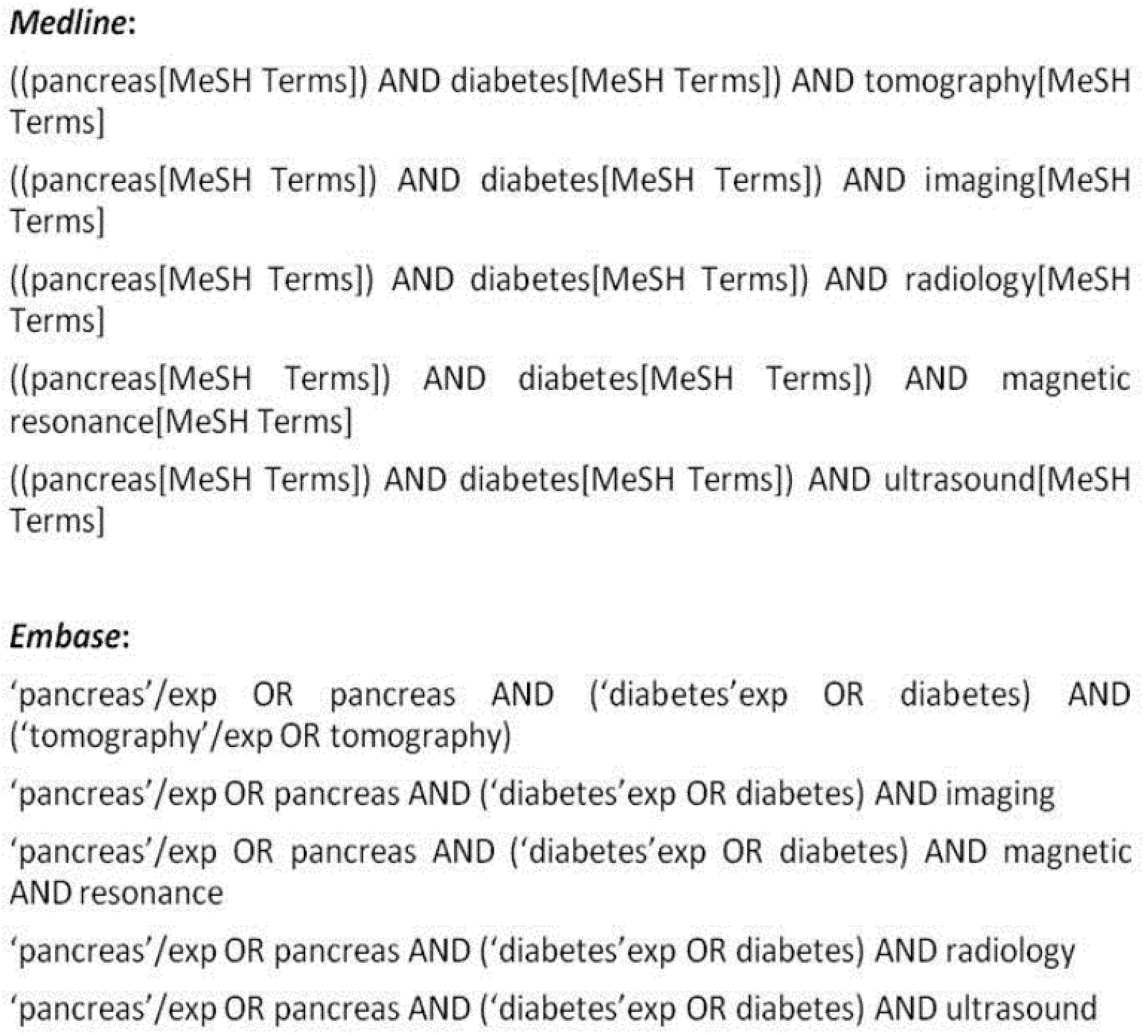
Search strategy used for study selection.

To identify observational studies evaluating pancreatic size or fat content by imaging in diabetes, the literature (Medline and Embase) was searched for studies using the three major imaging methods (US, CT, and MRI) for pancreas evaluation from inception until May 2017. No language or date restrictions were applied. Medical subject heading (MeSH) terms and key words included in the search were as follows: pancreas, diabetes, imaging, radiology, ultrasound, tomography, and magnetic resonance. Detailed Medline and Embase search strategies are shown as Fig 1. Also, the references of selected articles were manually searched. Titles and abstracts of all retrieved articles were independently reviewed by two physicians, T.S.G (radiologist) and T.H.R. Disagreements were resolved by consensus. The full text of selected articles was examined. The study is registered at PROSPERO under the number 2016: CRD42016039853.

### Study selection

We included prospective and retrospective observational studies evaluating pancreatic size and/or fat content by US, CT, or MRI in T1DM and/or T2DM patients as compared to non-diabetic subjects. Exclusion criteria were as follows: case report design, inclusion of pediatric patients (<12 years), absence of control group, and no clear description of imaging and post-processing technique. If duplicate studies were detected, the most complete report with the longest follow-up was included.

### Data extraction

One reviewer (T.S.G) conducted data extraction and a second investigator checked data extraction for accuracy (T.H.R). Data were extracted on year of publication, number of T1DM, T2DM, and non-diabetic subjects, and imaging method used for pancreatic assessment. In addition, the following data were extracted on pancreatic parameters (mean and standard deviation) in the three groups of interest (T1DM, T2DM, and non-diabetic subjects): pancreatic diameter in cm, area in cm^2^, volume in cm^3^, density in Hounsfield units (HU), and percentage of pancreatic fat.

### Quality assessment

Quality assessment of studies included in meta-analyses was performed using the Newcastle-Ottawa scale [26], a scoring system that takes into account selection of groups, comparability between groups, and ascertainment of the exposure (in the case of studies included in this meta-analysis, it refers to the imaging method and to the technique by which parameters were measured).

### Data synthesis and analysis

Absolute changes in size (diameter, area, or volume), density, and fat percentage in patients with diabetes and control groups are presented as means ± standard deviation (SD). Cochran's Q test was used to evaluate heterogeneity between studies. A p value <0.1 was considered statistically significant. The *I*^*2*^ test was also conducted to evaluate the magnitude of the heterogeneity between studies. Heterogeneity was defined as *I*^*2*^>50%. A random effects model was used for all analyses.

The contribution of individual studies to the overall heterogeneity was explored using meta-regression, subgroup analyses, and sensitivity analyses by removing each study at a time and re-running the meta-analyses. In some cases, these procedures were not feasible due to an insufficient number of studies/patients.

Funnel plot asymmetry was evaluated by Begg and Egger tests. The impact of small-study bias was considered as significant if p value <0.1 [27]. Analyses were conducted using Stata software version 11.0 (Stata Inc, College Station, Texas).

## Results

A total of 5,634 potentially relevant studies were initially identified, 1,532 in Medline and 4,102 in Embase. Hand search of reference lists resulted in the inclusion of an additional eight articles (5,642 articles). After removal of 1,047 duplicates, 4,595 citations were screened based on titles and abstracts. Twenty-nine were selected for full-text review, and finally 23 articles and one poster fulfilled the inclusion criteria.

**Fig 2:**
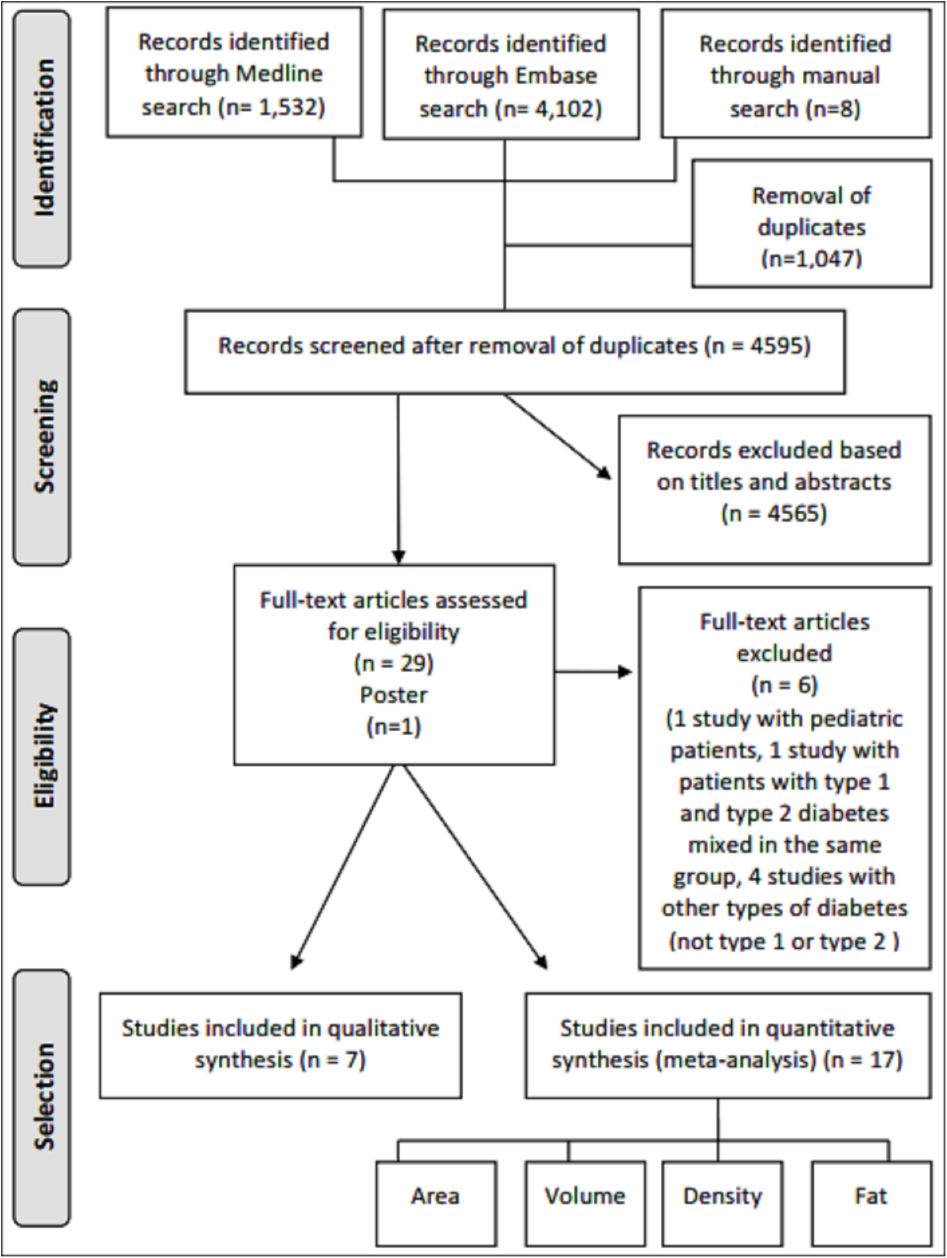
Flowchart of study selection.

Of the 24 studies selected, seven were not included in the meta-analysis: in six, data were not extractable [12, 24, 28–31], and in one study measuring pancreatic fat divided by splenic fat the parameters of interest could not be combined[23]. The remaining 17 studies were included in meta-analyses: two evaluating diameter [19, 32], two evaluating area[16, 33]eight evaluating volume [17, 18, 20, 34–38], two evaluating density [17, 39], and six evaluating fat content [17, 18, 40–43]. Studies assessing multiple parameters were included in more than one meta-analysis. The flowchart of study selection is depicted in Fig 2.

Characteristics of the studies included in the systematic review and meta-analyses are presented in Table 1. The studies were published between 1985 and 2015 and included 3,403 participants: 284 T1DM patients (minimum-maximum: 12 to 60 patients), 1,139 T2DM patients (11 to 198 patients), and 1,980 control subjects (9 to 660 patients). Overall mean age was 59.4 years (minimum-maximum: 13 to 100 years) (33.9 in T1DM, 58.1 in T2DM, and 63.0 years in controls), and overall mean BMI was 26.84 kg/m^2^ (22.72 kg/m^2^ in T1DM, 27.05 kg/m^2^ in T2DM, and 27.21 kg/m^2^ in controls). Mean duration of disease was 8.9 years in T1DM and 6.5 years in T2DM. CT scanners used for evaluation of pancreatic volume ranged from one to 64-detector-rows. MRI evaluating pancreatic volume and/or pancreatic fat percentage was performed in 1.5 T or 3.0 T scanners. In both CT and MRI scans, the contour of the pancreas in each slice was annotated, automatically generating the area of each slice. Pancreatic volume was then calculated by multiplying the area of pancreatic tissue on each section by the interval between slices. MRI methods for estimation of pancreatic fat content included spectroscopy or chemical shift sequences for separation of water and fat. CT quantification of pancreatic fat was performed by means of a histogram.

**Table 1:** Summary of studies evaluating pancreas size and fat content by imaging methods in diabetes.

	No. of subjects					Results		
Authors, year	Type 1 diabetes	Type 2 diabetes	Controls	Method	Parameter	Type 1 diabetes	Type 2 diabetes	Controls
Fonseca et al, 1985 [28]	32	22	19	US	Area	-	-	-
Silva et al, 1993* [19]	36	40	60	US	Diameter	1.9±0.3^#^ 0.9±0.2^§^	2.7±0.4 1.2±0.3	2.4±0.4 1.1±0.3
Alzaid et al, 1993* [16]	43	14	19	US	Area	10.2±3.0	-	15.0±2.1
Rajput et al, 2001* [33]	0	35	15	US	Area	10.4±4.61	-	16.59±2.49
Basiratnia et al, 2007* [32]	60	60	60	US	Diameter	1.72±0.28^#^ 0.79±0.16^§^	2.09±0.36 0.94±0.21	2.42±0.40 1.35±0.21
Gilbeau et al, 1992* [39]	37	20	57	CT	Density	-	37.62±15.14	40.00±15.10
Goda et al, 2001* [20]	29	26	22	CT	Volume	45.2±19.5	68.7±18.8	71.5±18.8
Phillipe et al, 2001 [12]	28	24	0	CT	Volume	-	-	-
Saisho et al,2007* [35]	165	0	660	CT	Volume	-	70.0±26.5	74.9±27.0
Yokota et al, 2012 [29]	62	0	53	CT	Density	-	-	-
Lim et al, 2014* [17]	0	156	50	CT	Volume, density, fat%	-	V: 53.8±13.4 D: 49.39±5.84 F: 6.7±6.1	66.3±13.9 54.60±5.80 2.9±3.4
Kim et al, 2014 [30]	18	0	33	CT	p-s, p/s	-	-	-
Kim et al, 2014 [23]	198	0	0	CT	Density	-	-	-
Tushuizen et al, 2007 [24]	12	0	24	MRI	Fat%	-	-	-
Williams et al, 2007* [34]	0	12	12	MRI	Volume	52.4±17.1	-	101.0±19.5
Sequeiros et al, 2010* [36]	0	12	12	MRI	Volume	52.5±19.6	-	104.8±21.8
Lim et al, 2011* [40]	11	0	9	MRI	Fat% (c)	-	8.0±5.3	6.0±3.9
Williams et al, 2012 [37]	0	19	24	MRI	Volume	91.9±28.6	-	121.3±31.8
Burute et al, 2014* [38]	32	0	50	MRI	Volume	-	72.7±20.7	89.6±22.7
Ma et al, 2014* [41]	24	0	10	MRI	Fat% (s)	-	18.2±12.5	6.9±1.6
Percival et al, 2014* [43]	71	0	9	MRI	Fat% (c)	-	5.5±22.2	4.9±12.5
Macauley et al, 2015* [18]	41	0	14	MRI	Volume, fat% (c)	-	V: 55.5±17.9 F:5.4±1.9	82.6±17.9 4.4±1.5
Kuhn et al, 2015* [42]	0	60	45	MRI	Fat% (c)	-	4.6±7.7	4.4±5.3
Begovatz et al, 2015 [31]	14	0	28	MRI	Fat%	-	-	-

US: ultrasound; CT: computed tomography; MRI: magnetic resonance image;fat% (s): pancreatic fat percentage obtained by MRI spectroscopy; fat% (c): pancreatic fat% obtained by MRI chemical shift imaging.

P-S: difference between pancreatic and splenic density; P/S: pancreas-to-spleen density ratio.

V: volume; D: density; F: fat%

*: studies included in meta-analyses.

^#^:diameter of pancreatic head.

^§^:diameter of pancreatic body.

Diameter is shown in cm; area in cm^2^; volume in cm^3^; density in HU; fat in %.

Results are shown as mean±SD.

### Pancreatic size

#### Volume

**Fig 3:**
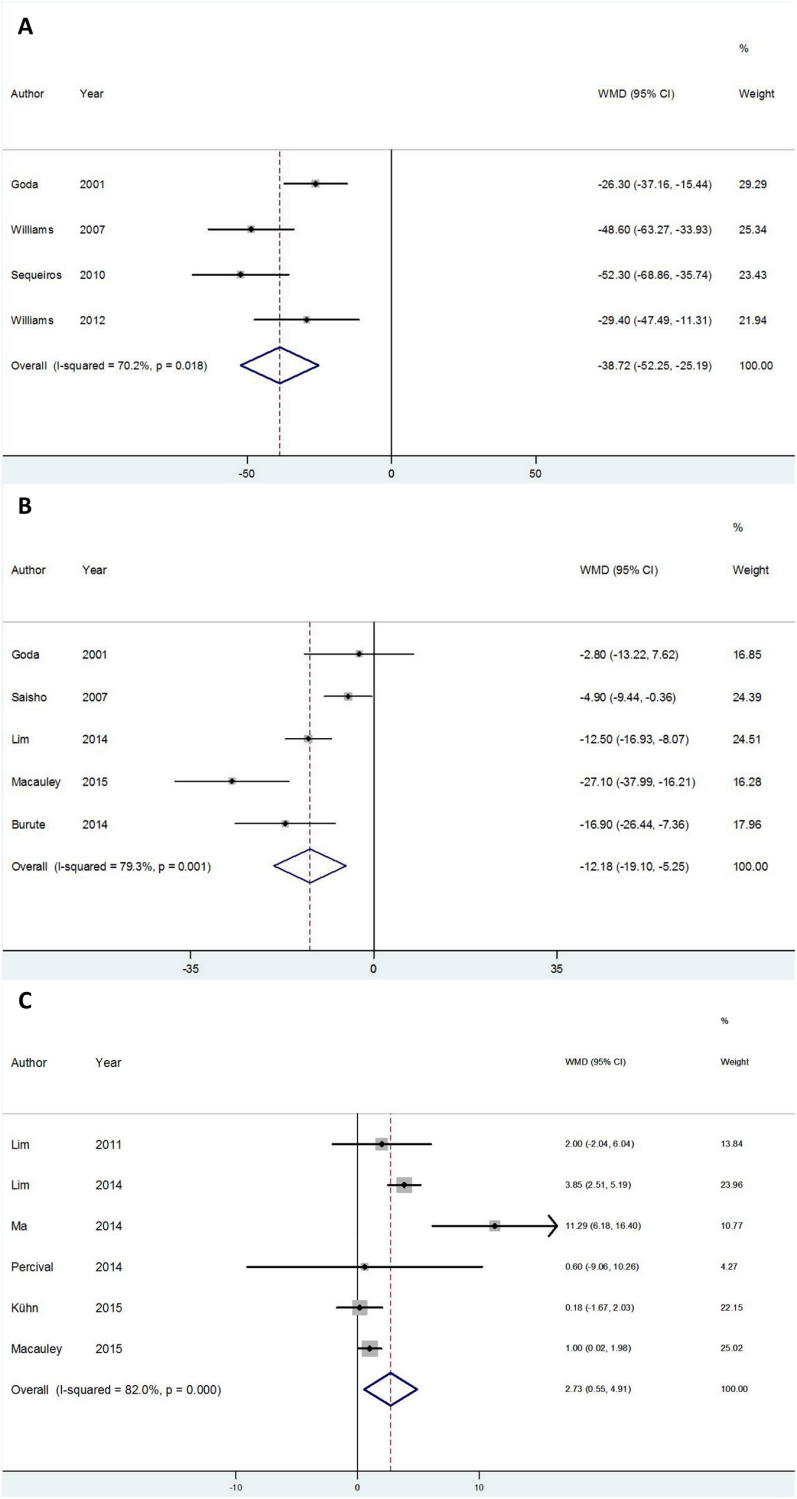
Meta-analyses of studies evaluating pancreas by imaging in diabetes. (A) Forest plot comparing pancreas volume (cm^3^) in type 1 diabetic patients with a control group. (B) Forest plot comparing pancreas volume (cm^3^) in type 2 diabetic patients with a control group. (C) Forest plot comparing fat content (%) in type 2 diabetic patients with a control group. WMD = weighted mean difference.

Eight studies were included in meta-analyses focusing on volume[17, 18, 20, 34–38]. In four studies[20, 34, 36, 37] with T1DM patients, pancreas volume was reduced as compared to control subjects (–38.72 cm^3^; 95%CI –52.25 to –25.19). However, between-study heterogeneity was high (*I*^*2*^ = 70.2%, p for heterogeneity = 0.018) (Fig 3A).

Heterogeneity was explored by sensitivity analysis and each study was excluded at a time. Heterogeneity was reduced to 47.8% (p for heterogeneity = 0.147) after omission of the study by Goda *et al* [20], while no change was observed when other studies were excluded. This may have resulted from patient selection bias, as the mean age of T1DM patients in this study was 48.7 years, while the mean duration of diabetes was 9.4 years–suggesting that T2DM patients may have been misdiagnosed withT1DM. Interestingly, this was the only study using CT for volume assessment, and thus sensitivity and subgroup analysis of pancreatic volume based on image technique were coincident; in this study, pancreatic volume in T1DM patients was 26.3 cm^3^ smaller than that of controls (95%CI, -37.16 to -15.44). Moreover, the present meta-analysis of MRI studies shows a mean reduction of -44.08 cm^3^ (95%CI -57.16 to -30.99) in pancreatic volume in T1DM patients *vs*. controls. Subgroup analyses were performed based on the quality of studies (including only studies with a score of 6–8 in the Newcastle-Ottawa scale or studies where cases and controls were matched by BMI. However, heterogeneity was not affected by these variables (data not shown).

**Fig 4:**
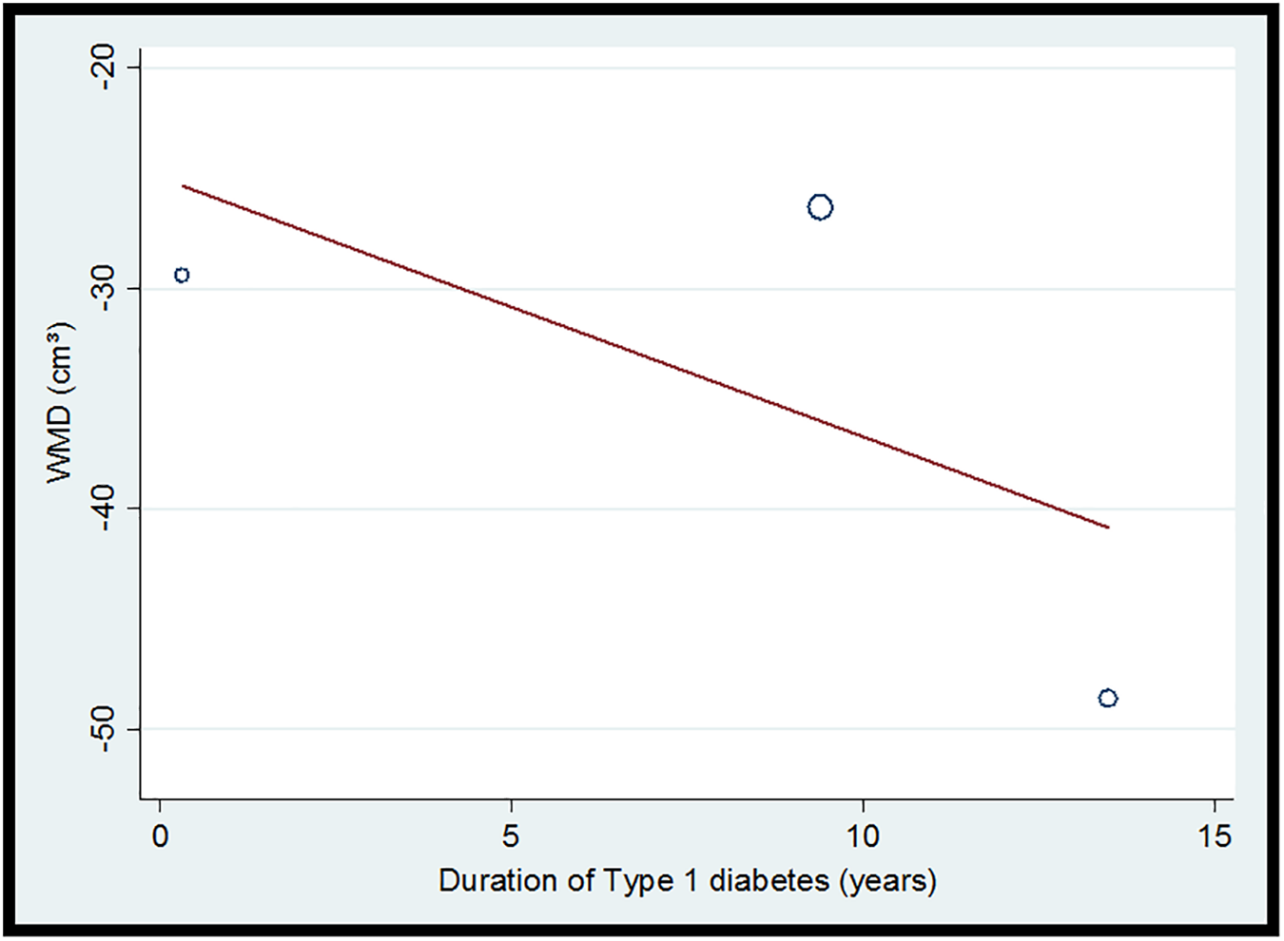
Bubble plot of the relation between diabetes duration (years) and pancreatic volume (cm^3^) in type 1 diabetic patients, including references [20, 34, 37]. WMD = weighted mean difference.

Despite the small number of studies, we performed meta-regression with age, BMI, and duration of diabetes as covariates. Although not statistically significant, a reduction in heterogeneity from 70.2% to 52.76% (p = 0.355) was observed in the model considering duration of diabetes. Interestingly, the T1DM patients with longer diabetes duration had the lowest pancreatic volume (Fig 4).

Similar results were observed for pancreatic volume in five studies[17, 18, 20, 35, 38] with T2DM patients, whose pancreas was smaller than that of controls (-12.18 cm^3^; 95%CI: -19.1 to -5.25, *I*^*2*^ = 79.3%, p for heterogeneity = 0.001) (Fig 3B). Sensitivity analysis excluding individual studies did not decrease heterogeneity (data not shown). However, subgroup analysis considering imaging methods showed lower heterogeneity for MRI studies (*I*^*2*^ = 47.6%, p for heterogeneity = 0.167) in comparison with CT studies (*I*^*2*^ = 70%, p for heterogeneity = 0.035). It should be noted that pancreas volume was smaller in T2DM patients *vs*. controls regardless of imaging technique (MRI: -21.65 cm^3^ [95%CI -31.62 to -11.68] and CT: -7.5 cm^3^ [95%CI -13.65 to -1.36]). As for T1DM, subgroup analyses considering only studies rated 6–8 in the Newcastle-Ottawa scale or studies with BMI-matched T2DM patients did not change heterogeneity (data not shown). No variable included in meta-regression was associated with heterogeneity.

Only one study [12] compared pancreas volume inT1DM and T2DM patients, precluding meta-analysis. In this CT study, no significant differences in volume were detected between T1DM and T2DM patients.

#### Diameter

Two studies[19, 32] using US detected a smaller pancreatic diameter (measured at the head and body) in T1DM patients as compared to controls (head diameter: –0.6 cm [95%CI: –0.8 to –0.41], *I*^*2*^ = 77.8%, p for heterogeneity = 0.034; and body diameter: –0.38 cm [95%CI: –0.73 to –0.03], *I*^*2*^ = 97.2%, p for heterogeneity<0.001). However, no differences were found when T2DM patients and controls were compared (head diameter: –0.02 cm [95%CI: –0.63 to 0.6], *I*^*2*^ = 97%, p for heterogeneity<0.001; and body diameter: –0.16 cm [95%CI: –0.66 to 0.34], *I*^*2*^ = 97.9%, p for heterogeneity<0.001).

A meta-analysis comparing pancreas diameter in T1DM and T2DM revealed that both head diameter (–0.58 cm [95%CI: –1 to –0.16], *I*^*2*^ = 94.5%, p for heterogeneity<0.001) and body diameter (–0.22 cm [95%CI: –0.36 to –0.07], *I*^*2*^ = 79.3%, p for heterogeneity = 0.028) were smaller in T1DM patients.

#### Area

Two US studies [16, 33]analyzed T1DM patients regarding pancreatic area, which was significantly smaller as compared to that of controls (-5.44 cm^2^ [95%CI: –6.8 to –4.08], *I*^*2*^ = 1.9%, p for heterogeneity = 0.313). Another study [28], which was not meta-analyzed due to lack of extractable data, corroborated these findings, showing reduced pancreas area in T2DM, and especially T1DM patients.

### Pancreatic fat content

Six studies[17, 18, 40–43] including only T2DM patients evaluated pancreatic fat content in terms of fat percentage, which was higher in T2DM patients as compared to control subjects (+2.73% [95%CI: 0.55 to 4.91], *I*^*2*^ = 82.0%, P for heterogeneity<0.001) (Fig 3C). Heterogeneity was not explained by either sensitivity analysis/meta-regression (data not shown) or subgroup analysis based on imaging methods; only one study [17] measured pancreatic fat content by CT. Meta-analysis of the additional five studies [18, 40–43], all of which used MRI, did not change heterogeneity (*I*^*2*^ = 75.7%, p for heterogeneity = 0.002). Similarly, heterogeneity was unchanged in subgroup analyses of studies with Newcastle-Ottawa scores of 6–8 or of studies with BMI-matched groups (data not shown).

Pancreatic density is an indirect form of evaluating fat content, as fat-enriched tissues have lower densities. Pancreatic density assessed by CT in two studies [17, 39] was lower in T2DM patients *vs*. control subjects (–4.98HU [95%CI: –6.76 to –3.21], *I*^*2*^ = 0%, p for heterogeneity = 0.395).

Interestingly, Yokota *et al* demonstrated a decrease in pancreatic density with increasingly impaired glucose homeostasis[29]. Healthy individuals had higher pancreatic densities, which decreased progressively from impaired glucose tolerance to diabetes [29]. However, Begovatz *et al* did not find differences in pancreatic fat content between subjects with normal glucose, impaired fasting glucose, or T2DM patients when pancreatic fat was evaluated by MRI [31].

### Quality of studies and small-study bias

The studies included in meta-analyses were assessed for quality using the Newcastle-Ottawa scale (Table 2). Overall, studies had low/moderate quality; most had a score of 6 or 7 points from a maximum of 9.

**Table 2:** Newcastle-Ottawa quality assessment of studies included in meta-analyses.

	Selection				Comparability	Outcome			
Authors, year	1	2	3	4	5	6	7	8	Score
Gilbeau et al, 1992 [39]				*	** (age, diabetes duration)	*	*	*	6
Silva et al, 1993 [19]				*		*	*	*	4
Alzaid et al, 1993 [16]	*	*		*		*	*	*	6
Rajput et al, 2001 [33]	*			*	** (age, sex, BMI)	*	*	*	7
Goda et al, 2001 [20]		*		*	** (age, sex)	*	*	*	7
Basiratnia et al, 2007 [32]	*		*	*	** (age, sex)	*	*	*	8
Saisho et al, 2007 [35]	*	*		*	** (age, BMI)	*	*	*	8
Williams et al, 2007 [34]	*			*		*	*	*	5
Sequeiros et al, 2010 [36]	*			*	** (age, sex)	*	*	*	7
Lim et al, 2011 [40]	*			*	** (age, sex, weight)	*	*	*	7
Williams et al, 2012 [37]	*	*		*	** (age, weight)	*	*	*	8
Lim et al, 2014 [17]	*			*	** (age, BMI)		*	*	6
Burute et al, 2014 [38]	*	*		*	** (age, sex, weight)	*	*	*	8
Ma et al, 2014 [41]	*	*		*	* (age)		*	*	6
Percival et al, 2014 [43]	*			*		*		*	4
Kühn et al, 2015 [42]			*	*	** (age, sex, BMI)	*	*	*	7
Macauley et al, 2015 [18]	*			*	** (age, sex, weight)		*	*	6

The number of stars indicates the quality of each item evaluated: minimum 0, maximum 1 star for selection and outcome; and minimum 0, maximum 2 stars for comparability. The maximum possible overall score is 9.

The funnel plot asymmetry test revealed no major small-study bias regarding volume or fat content in T2DM patients (p = 0.458 and 0.484 respectively). However, a possible small-study bias was detected for volume in T1DM patients (p = 0.041).

## Discussion

In this systematic review with meta-analysis of imaging studies, a reduction in pancreatic size was observed in both T1DM and T2DM patients. In addition, an increase in pancreatic fat content was seen in T2DM subjects.

Pancreatic size was evaluated in terms of diameter, area, and volume. In T1DM patients, the results show decreased pancreatic size in comparison to non-diabetic controls for all three parameters. In turn, volume, but not diameter, was reduced in T2DM patients; area was not meta-analyzed because only one study focusing on this aspect included T2DM subjects. Interestingly, a comparison between T1DM and T2DM revealed smaller pancreatic diameter in T1DM individuals. A single study assessing pancreatic area showed smaller dimensions in T1DM individuals vs. T2DM individuals, and the only study assessing volume observed no differences between T1DM and T2DM patients.

Volume, which provides three-dimensional data, is the best parameter to assess organ size. The present findings show smaller pancreatic volume in both T1DM and T2DM patients in relation to controls, but data are insufficient to establish a firm conclusion regarding the comparison between these two types of diabetes. However, our meta-analyses focusing on volume suggest that T1DM subjects may in fact have smaller pancreatic volume in relation to T2DM individuals: a difference of –38.72 cm^3^ (95%CI –52.25 to –25.19) was observed for T1DM vs. controls, and a difference of –12.18 cm^3^ (95%CI –19.1 to –5.25) was observed for T2DM vs. controls. Although a formal statistical test was not performed, it is fair to assume that pancreatic volume was smaller in T1DM than T2DM patients, since the 95%CIs did not overlap.

An intriguing finding of this systematic review is the low heterogeneity of MRI studies, as opposed to the high heterogeneity of CT studies. This might be due to the fact that the MRI studies cover a shorter time interval and more recent years (2007–2015) as opposed to the CT studies (1992–2014). Moreover, the magnitude of volume reduction detected by each imaging method was remarkably different (T1DM: –44.08 cm^3^ for MRI *vs*. –26.3 cm^3^for CT; T2DM:–21.65 cm^3^ for MRI *vs*. *–*7.5cm^3^for CT), with MRI showing consistently higher differences in volumes between patients with diabetes and controlsthan the results obtained by CT. These differences were unexpected, since the tool used to measure pancreatic volume is similar in both imaging methods and no plausible technical reason can justify lower volumes measured by MRI. Furthermore, a recent study evaluating T1DM patients with MRI or CT did not report differences in pancreas size measured by the two methods [44].

There is a large inter-individual variation in pancreas morphology and volume related to body size and age in healthy populations [45, 46]. This may be a relevant source of confusion in studies with T1DM and T2DM individuals–T1DM patients are usually younger, and, as shown in the present study, possibly have a smaller pancreas; conversely, T2DM patients might be older than controls, and pancreas size may decrease with age [35]. However, most studies in the present review included BMI-and age-matched controls, and neither subgroup analysis nor meta-regression considering these possible confounders showed an impact on heterogeneity. Reduced pancreatic volume and weight are present from early phases of T1DM, as demonstrated by a study comparing the pancreasofT1DM donors and controls [14], even after correction for confounders [15]. Recently, Virostko *et al* [44] have suggested progressively smaller pancreatic volume with increased duration of T1DM (decline rate of 0.013 cm^3^/kg per year). This is supported by the findings of our meta-regression showing that TD1M patients with longer disease duration had lower pancreatic volumes. Thus, monitoring variations in pancreatic volume might be useful to predict diabetes in high-risk individuals [14]. Recently, Yun *et a l*have reported that pancreatic volume reduction rate calculated by serial CT volumetry is a significant predictor of new-onset diabetes in patients undergoing pancreaticoduodenectomy[47].

Pancreatic fat content is evaluated by means of density or fat percentage, with percentage being more precise. Some studies suggest an association between increased pancreatic fat and diabetes. Kim *et al* [23] have shown that two CT indexes–the difference between pancreatic and splenic density and the pancreas to spleen density ratio–are higher in patients with impaired glucose tolerance. In line with this, a study designed to compare pancreatic fat content and beta cell function found increased lipid deposition in the pancreas of diabetic patients as compared to healthy subjects [24]. Furthermore, in T2DM patients, obesity was associated with lower pancreatic density evaluated by CT, indicating higher pancreatic fat content [30]. In reverse order, return of normal insulin secretion and reduction of pancreatic fat content has been demonstrated after eight weeks of a low energy intake diet [40]. Our data indicate that pancreatic fat content is increased in T2DM patients, which may reflect a paracrine effect of insulin. Insulin resistance causes increased insulin secretion by beta cells, and the higher local insulin concentration may induce fat deposition. A similar phenomenon occurs in the liver when pancreatic islets are transplanted into the portal vein [48–50]. Pancreatic islets delivered to the hepatic sinusoids engraft and produce insulin, and focal steatosis is observed in 20% to 60% of islet recipients [48–50]. More interestingly, transplanted islets surrounded by fat have reduced function, probably as a result of lipotoxicity [50, 51]. Conversely, a low-fat diet and leptin overexpression have been shown to reduce fat content around islets, improving islet function in an animal model [51]. Taken together, these findings suggest that pancreatic fat accumulation might be a result of the higher local insulin levels in an insulin-resistant environment, and that pancreatic lipid deposition may further impair islet function.

Our results have some practical implications. First, the finding of a small or fatty pancreas using imaging techniques should prompt a recommendation for proper biochemical investigation of diabetes. Second, as there is some evidence in the literature linking pancreas atrophy in T1DM and T2DM patients with pancreatic exocrine deficiency [12, 13], differential diagnosis of chronic diarrhea in diabetic patients should consider exocrine pancreatopathy, a hypothesis that could be corroborated by diagnostic imaging.Important to say, ultrasonographic evaluation of the pancreas is not as accurate as evaluation by CT or MRI.

The present review has limitations that must be addressed. First, there are few studies assessing each parameter, precluding adequate exploration of heterogeneity and increasing the risk for small-study bias. Secondly, CT and MRI have different contrast mechanisms to quantify pancreatic fat content, and both lack detail in measurement methodologies. However, MRI is considered to be more reliable. Pancreatic fat measurement by CT is based on a histogram analysis in which anarbitrary threshold of -190 to -30 HU is applied to identify fat containing voxels [17], whileMRI uses spectroscopy or chemical shift techniques for this purpose [42]. These differencesmay explain discrepancies that can possibly be found between CT and MRI regarding pancreatic fat calculation.Thirdly, pediatric patients were excluded, although T1DM has an early age of incidence. As pancreatic volume is constitutionally associated with body size, and children have a relatively small body size, we think it would be better not to compare children with the adult population. Additionally, the overall quality of studies ranged from low to moderate. Furthermore, the studies included in the analysis date from 1985 to 2015, a time interval in which dramatic changes happened in imaging techniques, which might possibly influence the results of volume/area determinations.However, we believe that the findings of increased fat content and decreased pancreas size consistently point in the same direction and should not be dismissed.

In summary, the present data indicate that reduced pancreas size and increased fat content are features of diabetes. Further longitudinal studies are required to elucidate the cause and effect relationship between pancreatic size and diabetes, as well as the possible causes of pancreas shrinkage and fat deposition. A better understanding of the mechanisms of altered pancreas morphology and fat deposition in diabetes may lead to new insights in preventing, predicting, and treating patients with diabetes.

## Supporting information

S1 File PRISMA checklist. (DOCX)
